# Anticancer Activity of Thiophene Carboxamide Derivatives as CA-4 Biomimetics: Synthesis, Biological Potency, 3D Spheroid Model, and Molecular Dynamics Simulation

**DOI:** 10.3390/biomimetics7040247

**Published:** 2022-12-17

**Authors:** Mohammed Hawash, Mohammed T. Qaoud, Nidal Jaradat, Samer Abdallah, Shahd Issa, Nawal Adnan, Marah Hoshya, Shorooq Sobuh, Zafer Hawash

**Affiliations:** 1 Department of Pharmacy, Faculty of Medicine and Health Sciences, An-Najah National University, Nablus 00970, Palestine; 2 Department of Pharmaceutical Chemistry, Faculty of Pharmacy, Gazi University, Etiler, 06330 Ankara, Turkey; 3 Department of Biology & Biotechnology, Faculty of Science, An-Najah National University, Nablus 00970, Palestine; 4 Department of Biomedical Sciences, Physiology, Pharmacology & Toxicology Division, Faculty of Medicine and Health Sciences, An-Najah National University, Nablus 00970, Palestine; 5 Department of Physics, Faculty of Science, Birzeit University, Birzeit, Ramallah 71939, Palestine

**Keywords:** cancer, biomimetics, polar surface, 3D, thiophene, doxorubicin

## Abstract

The present study aimed to synthesize thiophene carboxamide derivatives, which are considered biomimetics of the anticancer medication Combretastatin A-4 (CA-4), and compare the similarity in the polar surface area (PSA) between the novel series and CA-4. Our results showed that the PSA of the most synthesized structures was biomimetic to CA-4, and similar chemical and biological properties were observed against Hep3B cancer cell line. Among the synthesized series **2b** and **2e** compounds were the most active molecules on Hep3B (IC_50_ = 5.46 and 12.58 µM, respectively). The 3D results revealed that both **2b** and **2e** structures confuse the surface of Hep3B cancer cell lines’ spheroid formation and force these cells to aggregate into a globular-shaped spheroid. The **2b** and **2e** showed a comparable interaction pattern to that observed for CA-4 and colchicine within the tubulin-colchicine-binding pocket. The thiophene ring, due to holding a high aromaticity character, participated critically in that observed interaction profile and showed additional advanced interactions over CA-4. The **2b** and **2e** tubulin complexes showed optimal dynamics trajectories within a time scale of 100 ns at 300 K temperature, which asserts their high stability and compactness. Together, these findings revealed the biomimetic role of **2b** and **2e** compounds in CA-4 in preventing cancer progression.

## 1. Introduction

Over the last two decades, globally, cancer has been one of the leading causes of death [1,2]. Liver cancer is regarded as one of the worst malignancies in the world., and its prevalence has increased in recent years. Hepatocellular carcinoma (HCC) is the most prevalent histologic type of liver cancer, accounting for the great majority of diagnosis and fatalities [3]. 

Chemotherapy is still the main method of cancer therapy for most common cancers, and usually it is used in combination with another strategy of cancer treatment [2,4]. In the last decade, a lot of research has aimed to innovate and develop novel chemotherapeutic agents by making modifications to the present [5,6], or by isolating chemical compounds from plants [7].

Several heterocyclic-containing compounds have antiproliferative activity [8,9], such as oxazoles [10,11] pyrazole [12,13,14,15,16], thiazole [17], indole [18,19], and thiophene [20]. Thiophene, a thiogenic heterocyclic scaffold, is one of the well-investigated chemical scaffolds for the innovation of novel compounds, with potential antiproliferative properties [21]. Heterocycles that have a sulfur atom are considered one of the most serious classes among other compounds that are usually applied in the chemistry field [22]. Thiophene-containing compounds have remarkable applications in different biological targets and their derivatives show analgesic and anti-inflammatory [23], antimicrobial [24], antihypertensive [25], and antitumor activities [26]. 

**Figure 1:**
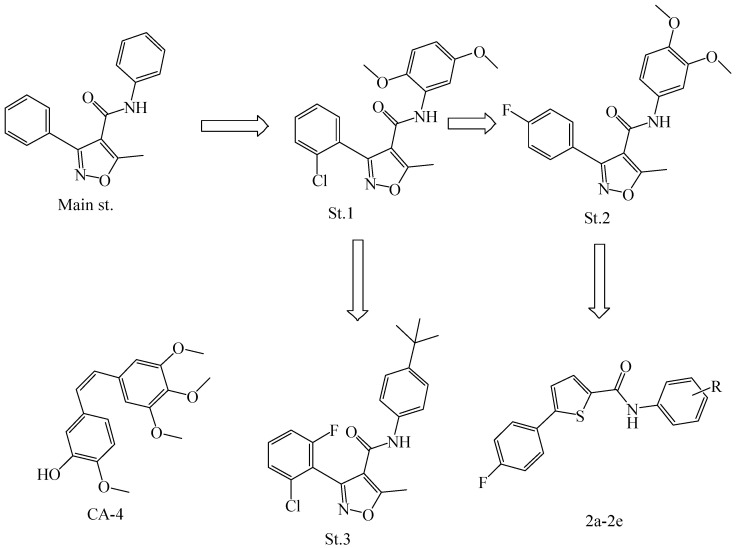
Chemical structures of CA-4, isoxazole-carboxamide series, and thiophene-carboxamide new series.

In the last 3 years, our team has aimed to synthesize a library of heterocyclic carboxamide derivatives that should have a trimethoxyphenyl moiety or similar substituents as promising anticancer agents that are biomimetic to combretastatin A-4 (CA-4). CA-4 (Figure 1) was extracted from *Combretum caffrum* plant [27,28] and exhibited significant anticancer properties by attaching to the colchicine structure site and entered clinical trial phases (II & III) [29,30]. However, CA-4 has several pharmacokinetic disadvantages including poor water solubility [31,32,33], in addition to its short plasma half-life and chemical instability [34,35]. 

About three different series were synthesized, and significant anticancer activities were observed. The main chemical structure is shown in Figure 1. Among the synthesized series, 3-(2-chlorophenyl)-N-(2,5-dimethoxyphenyl)-5-methylisoxazole-4-carboxamide (**St.1**; Figure 1) demonstrated significant activity against Hep3B (HCC) with an IC_50_ value of 23 µg/mL and cell cycle arrest in the G2/M phase [5]. After that, we aimed to change the position of the halogen from the ortho to para position, and the activities against Hep3B were raised with IC_50_ values less than 11.6 μg/mL for five compounds in this series, and one of the most active compounds was N-(3,4-dimethoxyphenyl)-3-(4-fluorophenyl)-5-methylisoxazole-4-carboxamide (**St.2**; Figure 1) with an IC_50_ value of 7.66 μg/mL [36]. Another series was synthesized with two halogens at ortho positions of phenyl, and the activities against Hep3B were improved. Among this series, *N*-(4-(*tert*-butyl)phenyl)-3-(2-chloro-6-fluorophenyl)-5-methylisoxazole-4-carboxamide (**St.3**; Figure 1) showed potent activators with an IC_50_ value of 2.77 µg/mL at the same tumor cell line [33].

The goal of this study is to synthesize a bunch of different series of similar structures, and the isoxazole heterocycle of previous works was replaced with a thiophene heterocycle, which results in new thiophene-carboxamide derivatives (**2a**–**2e**). Then they were chemically characterized by utilizing nuclear magnetic resonance (NMR) and high-resolution mass spectroscopy, determining their anti-proliferative property on several sorts of cancer cell lines by MTS assay, and the 3D Spheroid Model was performed to evaluate the tumor-stromal interaction on the bio-surface of Hep3B cell lines for the most active compounds. Furthermore, bioinformatics analysis was conducted to explain the recorded biological potency of the most potent compounds by molecular docking, dynamic studies, density functional theory (DFT) analysis, and ligand-based ADME and toxicity prediction.

## 2. Materials and Methods

### 2.1. Chemistry

All reagents and chemicals were brought from Sigma-Aldrich (Schnelldorf, Germany) and Alfa Aesar (Ward Hill, MA, USA). The Melting points were collected by utilizing the SMP-II Digital Melting Point instrument. Nuclear magnetic resonance spectroscopy (NMR) for ^1^H and ^13^C atoms were conducted on a Bruker instrument (500 MHz-Avance III High-Performance Digital) at the Faculty of Science, University of Jordan, Jordan. High-resolution mass spectroscopy was performed by utilizing a Waters LCT Premier XE Mass Spectrometer at Pharmacy Faculty, Gazi University, Ankara, Turkey.

#### General Procedure for the Synthesis of thiophene-Carboxamide (**2a**–**2e**)

In dichloromethane (DCM; 20 mL), 5-(4-fluorophenyl)thiophene-2-carboxylic acid (2.34 mmol) was dissolved and then DMAP (0.78 mmol) and EDC (3.12 mmol) were added and stirred under argon gas (inert gas) at room temperature for 30 min. After that, the appropriate aniline was added to the mixture and stirred for 48 hr. TLC monitored the reaction until it was completed. The excess aniline was removed by extraction with HCl. Under reduced pressure, the reaction mixture was dried using a rotarotavap. The residues of the obtained product were purified by column chromatography utilizing a DCM: ethyl acetate solvent system (1:1) [36,37].

### 2.2. Biological Methods

#### 2.2.1. Cytotoxicity Method

Several tumor cell lines were used in this study including HeLa (cervical adenocarcinoma) HepG2, and Hep3B (HCC); B16F1 (melanoma); MCF-7 (breast carcinoma); Colo205 (colon adenocarcinoma); and CaCo-2 (colorectal adenocarcinoma), in addition to two normal cell lines LX-2 (human hepatic stellate) and Hek293t (kidney cell lines). All evaluated cells were incubated in RPMI-1640 medium and supplemented by 1% of Penicillin/Streptomycin antibiotics, 1% of l-glutamine, and 10% fetal bovine serum. The selected cells were cultivated at 37 °C in a wet environment containing 5% CO_2_.. In a 96-well plate, the seeding of cells was conducted as 2.5 × 10^4^ cells per well. After 72 hr, the cells were confluent, the media was refreshed, and after that, all cells were incubated by several compounds’ concentrations (1, 10, 50, 100, and 300 µM). The viability of cells was investigated by the Cell Tilter 96^®^ Aqueous One Solution Cell Proliferation (MTS) assay regarding the manufacturer’s instructions (Promega Corporation, Madison, WI). In the last step, 20 μL of MTS per 100 μL of media were added to each well and kept at 37 °C; after that at 490 nm wavelength the absorbance was collected [38].

#### 2.2.2. Hepatocellular Carcinoma Spheroids Formation Test

Hep3B liver cancer cells (4 × 10^3^ per well) were seeded in round-bottomed supra-low attachment well plates over 24 h in the presence of **2b** or **2e** (17 μg/mL). Doxorubicin, a chemotherapeutic medication, was used as a positive control (100 μg/mL). Images of the formed spheroids/clusters were captured at 0 h and 24 h using an inverted microscope with a final magnification of 100×. The photographs of the spheroids were assessed and quantified using ImageJ software [39].

### 2.3. Bioinformatic Investigations

#### 2.3.1. Molecular Docking Studies

To estimate the binding pattern for the most active candidates of thiophene carboxamide derivatives within the tubulin-binding pocket (PDB ID: 6XER), the Maestro Schrödinger interface 12.3 was utilized to perform molecular docking studies [40]. The Tubulin protein was selected in this work because this protein is considered the main target of CA-4 and CA-4 biomimetics structures [19]. The PDB ID: 6XER, a 3D-crystallographic structure of tubulin in complex with colchicine, was selected as a target protein for docking simulation and dragged from Protein Data Bank (http://www.pdb.org, accessed on 10 October 2022). Firstly, the retrieved crude crystal structure was prepared using the Protein Preparation Wizard tool integrated into the Maestro interface. The preparation process involved pre-processing, retaining the co-crystallized ligand colchicine and the two essential chains (chain C and D), generating protonation and metal charge states, optimizing and removing water molecules beyond 3 Å of hits, and minimizing the structure at OPLS4 force field [41]. The target preparation step was followed by generating the receptor grid using the receptor grid generation module, and the 3D dimensions of the receptor grid were identified according to the co-crystallized ligand. The 2D-sketcher and Ligprep modules integrated into the maestro program were applied to draw and prepare the colchicine (reference candidate) and our newly designed thiophene carboxamide derivatives, respectively. The ligand preparation aimed to realize the ring conformations, stereochemistries, bond length, and bond angles. Moreover, it implicated the generation of their possible tautomers, ionization states at pH (7.0 ± 2.0) using Epik, chiralities, and lower energy conformations; in the end, they were minimized at OPLS4 force field [42].

At first, to validate the reliability of the docking protocol and software applied, the XP-Glide docking simulation was performed only on the cocrystallized ligand (colchicine). Then, the cocrystallized conformation was superposed on the docked conformation and the RMSD value was calculated. After that, the prepared ligands were docked to the target protein following the default protocol of the docking procedure. To have a better explanation of each binding pattern, the highest-ranked docked poses were subjected to the Protein–Ligand Interaction Profiler (PLIP) server and the interaction profiles were analyzed precisely [43].

#### 2.3.2. Free Energy Calculations Using Prime MM-GBSA

The Prime Molecular mechanics—Generalized Born Model and Solvent accessibility (Prime MM-GBSA) module, integrated into the maestro 12.3 interface, was utilized to estimate the binding energies of the docked ligands within the tubulin-binding site. Using the obtained XP-Glide pose viewer file of the docked ligands, the Prime MM-GBSA was run on the VSGB solvent model (The VSGB 2.0 Model, 2017) and OPLS4 force field and the calculated total free energy of each ligand-receptor complex was recorded. The change in free energy was calculated according to the following equation:

ΔG bind = G complex − (G protein + G ligand) (1)

where ΔG bind is the ligand-binding energy. G-complex, G-protein, and G-ligand are the minimized energies of the protein–ligand complex, unbound protein, and unbound ligand, respectively [44].

#### 2.3.3. Density Functional Theory (DFT) Analysis

The Jaguar-Single Point Energy module, integrated into the Maestro 12.3 interface, was used to perform the DFT analysis for the most active thiophene carboxamide derivatives. During that, some descriptors could be obtained such as atomic electrostatic potential (ESP) map surface, Lowest Unoccupied Molecular Orbital (E_LUMO_), Highest Occupied Molecular Orbitals (E_HOMO_), and HOMO-LUMO gap (ΔE) [45]. For the DFT analysis, the density theory and basic sets used were Yang–Parr correlation functional (B3LYP) and 6-31G**, respectively; the SCF spin treatment was set as automatic, medium grid density, quick accuracy level, and maximum iterations were set to be 48 [46,47].

#### 2.3.4. Molecular Dynamics (MD) Simulations

The online WEBGRO Macromolecular Simulations server (https://simlab.uams.edu/ProteinWithLigand/index.html; accessed on 20 October 2022) was used to perform the molecular dynamics simulations based on GROMAC. It is a public service that includes a GRACE High-Performance Computing Facility administrated by the University of Arkansas for Medical Sciences (UAMS) [48]. At first, the ligand topology was created utilizing the PRODRUG 2.3 online server (http://davapc1.bioch.dundee.ac.uk/cgi-bin/prodrg/submit.html; accessed on 1 October 2022) at GROMOS96 54a7 force field [49]. After that, the prepared prodrug structures and their related ligand–protein complexes were subjected to the protein with Ligand Simulation tool integrated into the WEBGRO server using the following input parameters: GROMOS96 54a7 force field, SPC water model for solvation, Triclinic box type, and Na+ and/or Cl^−^ ions were added as salt types to neutralize the system. The incorporated structures and complexes were energetically minimized using the steepest descent integrator to a max of 5000 steps. Regarding the equilibration and MD run parameters, the equilibration type was selected to be NVT/NPT at 300 K, 1.0 bar pressure, and leap-frog MD integrator type. For each system, the number of frames per MD simulation was fixed to 5000 and performed for a simulation of time 100 ns. As a result, various trajectories were obtained such as the radius of gyration (Rg) and the root-mean-square deviation (RMSD) used to estimate the formation and stability of complexes within the tubulin-binding pocket [50,51]. 

#### 2.3.5. Ligand-Based ADME/Toxicity Prediction

To assess the druggability of the tested thiophene carboxamide derivatives, a set of pharmacokinetics and physicochemical descriptors were calculated by utilizing the QikProp module integrated into the maestro Schrödinger suite 2021.3. This analysis explained many representative parameters that referred to absorption, distribution, metabolism, excretion, and toxicity (ADME-T) profiles of the tested structures. Thus, ADME-T analysis could highly estimate if the synthesized candidates potentially agree with the FDA-approved drugs in terms of their ADME-T profiles [52].

### 2.4. Statistical Analysis

All of the collected outcomes were expressed as mean ± SD standard deviation, and when the *p* value was <0.05, the result was considered significant.

## 3. Results

### 3.1. Chemistry

**Scheme 1:**
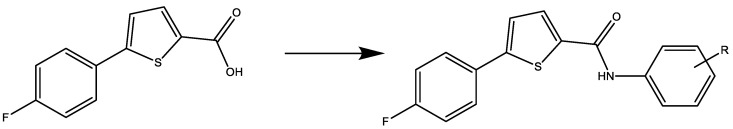
Acid and aniline derivatives were stirred in 15 mL of DCM, then DMAP and EDC were added under inert gas and stirred for 72 h.

The amide derivatives (**2a**–**2e**) were synthesized as shown in Scheme 1. The EDC was used as a coupling activator beside DMAP as a catalyst to synthesize carboxamide **2a**–**2e**; then the active species reacted with the aniline derivatives [53]. 

### 3.2. Chemical Characterization

*5-(4-fluorophenyl)-N-phenylthiophene-2-carboxamide* (**2a**)

Solid product M.P. 203.5–205 °C, HRMS (m/z): [M+H]^+^ Calc. 298.0702 found 298.0684. ^1^H NMR (DMSO-d_6_) δ: 10.25 (1H, s, NH), 8.03 (1H, d, *J* = 4 Hz, Ar-H), 7.80 (2H, t, J = 5 Hz, Ar-H), 7.75 (2H, d, J = 8 Hz, Ar-H), 7.60 (1H, d, J = 3.5 Hz, Ar-H), 7.37 (2H, t, *J* = 7.5 Hz, Ar-H), 7.31 (2H, t, *J* = 8.5 Hz, Ar-H), 7.12 (1H, t, *J* = 7.5 Hz, Ar-H). ^13^C NMR (DMSO-d6) δ ppm: 163.66, 161.70, 160.08, 147.66, 139.44, 139.12, 130.69, 130.13, 130.10, 129.15, 128.41, 128.34, 125.02, 124.25, 120.82, 116.76, 116.59.

*5-(4-fluorophenyl)-N-(3,4,5-trimethoxyphenyl) thiophene-2-carboxamide* (**2b**)

Solid product M.P. 190.8–192.0 °C, HRMS (m/z): [M+H]^+^ calc 388.1019 found 388.1073. ^1^H NMR (DMSO-d_6_) δ: 10.16 (1H, s, NH), 8.00 (1H, d, *J* = 3.5 Hz, Ar-H), 7.80 (2H, t, J = 5.5 Hz, Ar-H), 7.60 (1H, d, *J* = 4 Hz, Ar-H), 7.31 (2H, t, *J* = 9 Hz, Ar-H), 7.17 (2H, s, Ar-H), 3.79 (6H, s, 3,5-OCH_3_), 3.65 (3H, s, 4-OCH_3_). ^13^C NMR (DMSO-d6) δ ppm: 163.66, 161.70, 159.92, 153.11, 147.65, 139.49, 135.23, 134.25, 130.48, 128.39, 128.33, 125.05, 116.77, 116.59, 98.47, 60.57, 56.21.

*N-(4-(tert-butyl) phenyl)-5-(4-fluorophenyl) thiophene-2-carboxamide* (**2c**)

Solid product M.P. 218.5–220.4 °C, HRMS (m/z): [M+H]^+^ Calc. 354.1328, found 354.1347. ^1^H NMR (DMSO-d_6_) δ: 10.19 (1H, s, NH), 8.02 (1H, d, *J* = 4 Hz, Ar-H), 7.80 (2H, t, J = 7 Hz, Ar-H), 7.65 (2H, d, *J* = 8.5 Hz, Ar-H), 7.59 (1H, d, *J* = 3.5 Hz, Ar-H), 7.38 (2H, d, *J =* 8.5 Hz, Ar-H), 7.31 (2H, t, *J* = 8.5 Hz, Ar-H), 1.28 (9H, s, 4-t-butyl). ^13^C NMR (DMSO-d6) δ ppm: 163.64, 161.68, 159.93, 147.50, 146.58, 139.61, 136.54, 130.51, 130.16, 128.39, 125.77, 125.00, 120.56, 116.75, 34.53, 31.65

*N-(3,5-dimethoxyphenyl)-5-(4-fluorophenyl) thiophene-2-carboxamide* (**2d**)

Solid product M.P. 156.5–157.0 °C, HRMS (m/z): [M+H]^+^ Calc. 358.0913 found 358.0392. ^1^H NMR (DMSO-d_6_) δ: 10.16 (1H, s, NH), 8.02 (1H, d, *J* = 4 Hz, Ar-H), 7.80 (2H, t, *J* = 6 Hz, Ar-H), 7.60 (1H, d, *J* = 4 Hz, Ar-H), 7.31 (2H, t, *J* = 8.5 Hz, Ar-H), 7.03 (2H, s, Ar-H), 6.28 (1H, s, Ar-H), 3.75 (6H, s, 3,5-OCH_3_). ^13^C NMR (DMSO-d6) δ ppm: 163.68, 161.72, 160.88, 160.10, 147.80, 140.81, 139.39, 130.69, 130.09, 128.35, 125.04, 116.60, 98.93, 96.29, 92.82, 55.60.

*N-(4-chloro-2,5-dimethoxyphenyl)-5-(4-flurophenyl)thiophene-2-carboxamide* (**2e**)

Solid product M.P. 185.5–187.5 °C, HRMS (m/z): [M+H]^+^ Calc. 392.0588 found 358.0581. ^1^H NMR (DMSO-d_6_) δ: 9.64 (1H, s, NH), 8.02 (1H, d, *J* = 4 Hz, Ar-H), 7.80 (2H, t, *J* = 8.5 Hz, Ar-H), 7.58 (2H, d, *J* = 7 Hz, Ar-H), 7.31 (2H, t, *J* = 9 Hz, Ar-H), 7.22 (1H, s, Ar-H), 3.83, 3.81 (6H, s, 2,5-OCH_3_). ^13^C NMR (DMSO-d6) δ ppm: 160.06, 148.51, 147.77, 146.44, 138.77, 130.94, 128.45, 128.38, 126.24, 125.13, 117.61, 116.77, 116.59, 113.97, 110.54, 57.01, 56.96.

### 3.3. Biological Evaluations

#### 3.3.1. Cytotoxic Evaluation of the Compounds **2a**–**2e** MTS Assay Results

The MTS assay was done on B16-F1, Colo205, HepG2, Hep3B, CaCo-2, HeLa, and MCF7 cells, as well as the normal cell line Hek293t, to assess the antiproliferative effects of the synthesized compounds. As indicated in Table 1, five concentrations (300, 100, 50, 10, and 1 M) were used. According to Table 1, all chemicals have substantial antiproliferation properties against Hep3B cancer cell line, and significantly, compounds **2b**, **2d,** and **2e** were the most active agents with IC_50_ values of 5.46, 8.85, and 12.58 µM, respectively. Compounds **2b** and **2e** are considered the most potent compounds against most of the evaluated cancer cell lines; they have a very similar chemical surface that is a biomimetic to the CA-4 anticancer agent; in contrast, our synthesized molecules demonstrated weak or negligible effects on normal cell lines LX-2 and Hek293t.

According to various studies and works, carboxamides, polymethoxyphenyl, or alkyl-substituted compounds were effective anticancer agents [53,54]. This is because they have a similar or identical chemical moiety to CA-4 (trimethoxyphenyl), which means there is a similar polar surface area of the new compounds to CA-4, which makes these compounds able to permeate through the cell membrane and reach their target inside the cell, like tubulin.

#### 3.3.2. Dimensional (3D) Hepatocellular Spheroids’ Formation 

**Figure 2:**
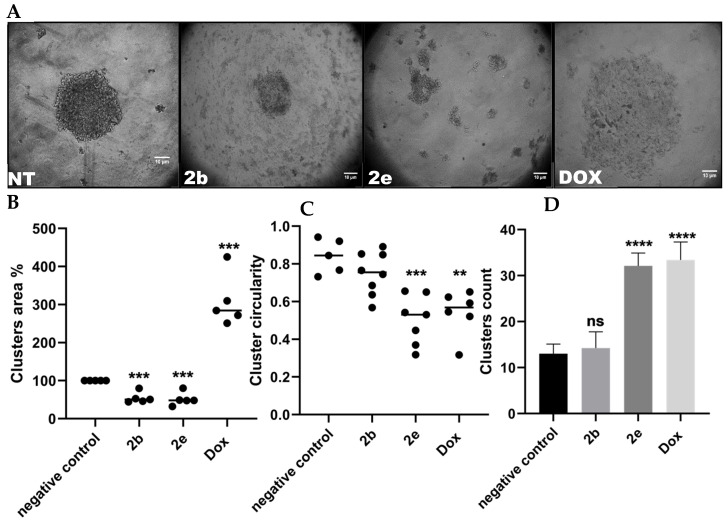
RHA-3 (**2b**) and RHA-6 (**2e**) perturb 3D hepatocellular spheroids’ formation. Images of cluster/s formed by Hep3B hepatocellular carcinoma in presence of **2b** (17 µg/mL) or **2e** (17 µg/mL) after 24 h of treatment compared to controls (**A**). Cluster percentage of occupied area relative to the negative control (**B**), cluster circularity (**C**), and cluster count (**D**). The non-treated cells are referred to as a negative control. At 100 μg/mL, DOX was utilized as a positive control. The scale bar represents a distance of 10 μm. Circularity scale: a value of 1 represents a perfect circle (ns: *p* > 0.05, **: *p* ≤ 0.01, ***: *p* ≤ 0.001, and ****: *p* ≤ 0.0001).

A growing body of research evidence supports the use of the Multicellular Tumor Spheroids (MCTS) system as a reliable 3D model for drug testing that mimics cancer tumor characteristics. For this reason, we looked at how the two most potent compounds **2b** and **2e** could affect the ability of Hep3B spheroids to form and their effect on the biosurface of these cancer cell lines. We demonstrated here that non-treated Hep3B cells formed a classically round large spheroid (MCTS). While **2b**-treated cells formed a significantly smaller spheroid (Figure 2A,B), 2e inhibited spheroid formation, instead forming a large number of smaller aggregates with distorted shapes (Figure 2A–D). In opposition, Hep3B cells formed huge flat cluster cells when incubated with Doxorubicin (positive control), with no recognized sphere formation (Figure 2A). These results showed that both **2b** and **2e** compounds perturb Hep3B spheroid formation and constrain cells to aggregate into a globular-shaped spheroid. Similarly, several previous reports showed that the inhibition of cancer spheroid formation in different cancer types is correlated with reduced cancer stemness, tumorigenicity, and chemotherapy resistance [55,56,57]. Moreover, these two compounds greatly decreased the size of the produced aggregates. Of note, there is a relationship between tumor size and sensitivity to chemotherapy; the latter is dependent on tumor size [58,59]. Together, these results suggest a potential role of **2b** and **2e** compounds in preventing cancer progression. 

### 3.4. Bioinformatics Studies

#### 3.4.1. Molecular Docking Simulations and MM-GBSA

**Figure 3:**
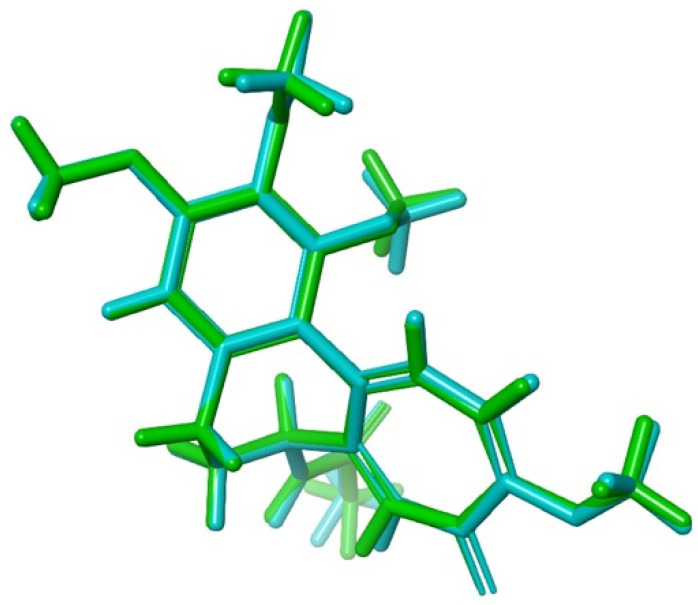
Superposition of the crystallized conformation (green) on the observed docked conformation (cyano) of the native ligand colchicine within tubulin protein structure (PDB ID: 6XER), RMSD value = 0.18 Å.

Aiming to explain the observed anti-cancer activity of **2b** and **2e** compounds, as CA-4 biomimetics showed a promising activity over the subjected cancer cell lines, the molecular docking simulation was performed within the colchicine-binding site of tubulin crystal structure (PDP ID: 6XER). Thus, the fitting orientation and ligand–protein-binding pattern inside the binding pocket were analyzed and compared to colchicine and CA-4 candidates, which were docked too as reference drugs. Out of 32 poses allowed to be generated for each ligand, the pose with the lowest XP-Glide-docking score was selected as the best to be discussed. At first, to evaluate the reliability of that retrieved tubulin crystal structure, docking protocol, and software utilized, the crystallized conformation of colchicine as a native ligand was superposed on the docked pose. Then as shown in Figure 3, the calculated RMSD value equaled 0.18 Ao, indicating that the protein structure, docking procedure, and facilities used are reasonable and valid. 

**Figure 4:**
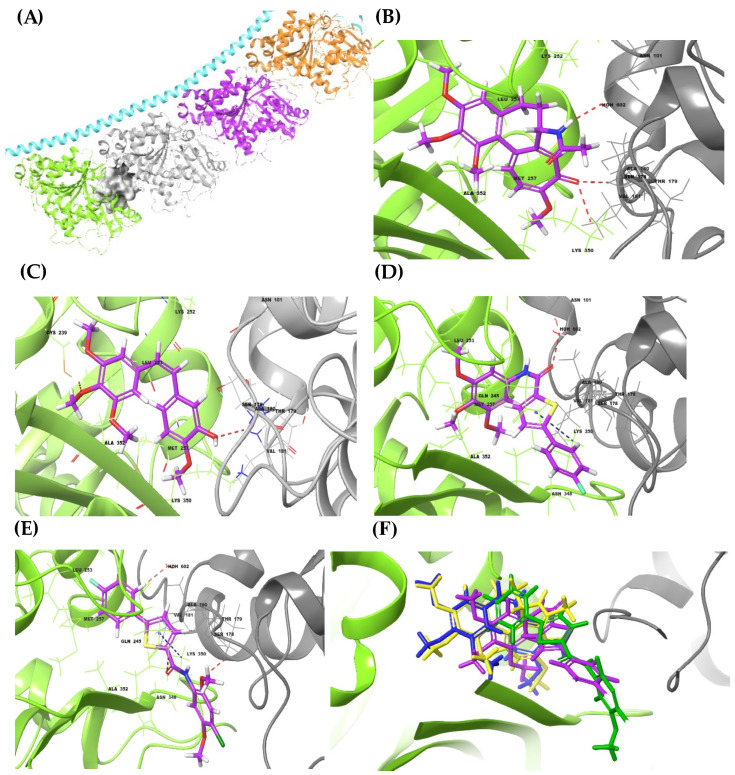
(**A**) The 3D-structure of tubulin protein (PDB ID: 6XER), chain A (orange), chain B (purple), chain C (gray), chain D (light green), and chain E (light blue); the colchicine-binding pocket is shown as a gray surface. (**B**–**E**) represent the molecular docking simulations of colchicine, CA-4 compound, **2b** compound, and **2e** compound within the tubulin-binding site. The hydrogen bond and π-cationic are represented by dashed red and blue colors, respectively. (**F**) represents the superposing of the docked poses of colchicine (yellow), CA-4 (blue), **2b** compound (purple), and **2e** compound (green).

The tubulin protein structure, as shown in Figure 4A, includes 5 chains (A, B, C, D, and E). The protein structure has two colchicine-binding sites, the one which was selected for our study exists as an interface between chains C and D (shown as a gray solid surface).

Docking of colchicine structure, as presented in Figure 4B, into the tubulin-colchicine-binding pocket, showed the best binding profile through the formation of two hydrogen bonds (HBs) with C-tubulin (V-181 and N-101) and one HBs with D-tubulin (K-350). This advanced interaction profile was also boosted with an additional five valuable hydrophobic interactions with D-tubulin that played a key role in fitting the colchicine structure deeper, so the tri-methoxy phenyl ring fits optimally within the binding site. Docking simulation of the CA-4 compound (Figure 4C), as a colchicine-analogous and well-known anti-cancer candidate, formed two HBs with V-181 and C-239 within C- and D-tubulin, respectively. Also, it accommodated deeply within the binding site as a result of forming an additional three hydrophobic interactions within D-tubulin. 

Analyzing the docking simulations of both thiophene Carboxamide derivatives (**2b** and **2e** compounds), as shown in Figure 4D,E, respectively, showed the formation of many advanced HBs and hydrophobic interactions within the binding pocket. **2b** Compound formed one HB with C-tubulin (N-101) and three hydrophobic interactions within D-tubulin, whilst **2e** Compound formed two HBs with C-tubulin (S-178) and D-tubulin (Q-245) and four hydrophobic interactions within D-chain. As observed, replacing the ethylene linker in the CA-4 structure with the aromatic thiophene ring resulted in incorporating an additional advanced interaction via the formation of π-cationic interaction between the thiophene ring and K-350 within D-chain compared to reference candidates. These observed poses of the four structures were merged in Figure 4F to explain the binding geometry more. As shown, the binding conformation of CA-4 is identical to that observed in colchicine and is simulated properly, which, besides its interaction profile, explains its high activity and potency. However, that modification in the linker scaffold led to an elongated conformation, which does not, like colchicine and CA-4, look like Cis-conformation, resulting in minimal potency to the references structures. As observed, the **2e** structure showed a better association and deeper submerging within the D-tubulin chain than **2b** structure, which explains its better anti-cancer activity over most tested cell lines. 

Thus, to boost the advanced contribution of the thiophene ring in their binding profiles and to optimize the structure geometry, it is suggested to just shift the position of the P-flour phenyl ring from position 5 to position 3, so that it can simulate the cis-conformational geometry observed in CA-4 and colchicine structures, which are expected to reveal better potency. 

All the calculated XP-Glide docking scores and MM-GBSA-binding energies regarding the reference candidates (colchicine and CA-4), **2b**, and **2e**-structures, are summarized in Table 2. As shown, the lowest docking score was recorded for colchicine (−9.23), then for CA-4 (−8.29), which is slightly better than that observed for **2e** (−6.46) and **2b** compounds (−6.13). The MM-GBSA values recorded to references, **2b**, and **2e** structures were also found in a close range, which is directly related to their docking scores, biology, and interaction profiles within the tubulin-colchicine-binding pocket.

#### 3.4.2. Density Functional Theory Analysis

**Figure 5:**
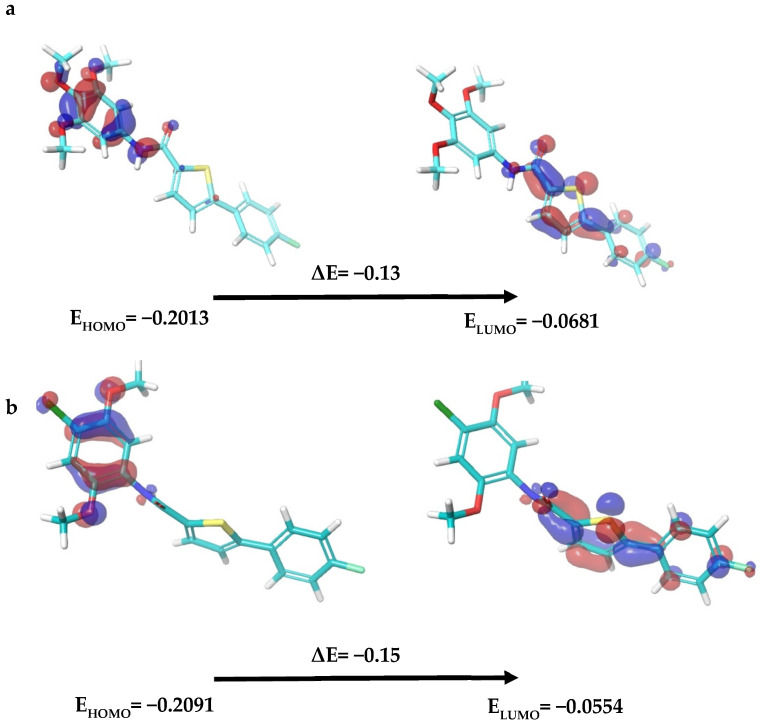
The 3D HOMO and LUMO profiles, the orbital energy values, and the energy gap (ΔE) of **2b** (**a**) and **2e** (**b**) molecules. The red and blue colors refer to positive lobes and negative lobes, respectively.

Based on the frontier orbital theory, the constituent shape and symmetry of the anti-bonding highest occupied molecular orbital (HOMO) and lowest unoccupied molecular orbital (LUMO) are certain features that play an influential role in modulating the chemical reactivity and stability of molecules inside the binding pocket of the target protein [60]. The antibonding HOMO and LUMO orbitals are molecular orbitals of high energy, referring to their high ability to donate and accept electrons, respectively. So the electron-rich HOMO orbitals are always associated with the electrophilic attack, while the electron-deficient LUMO orbitals make it an ideal target for the nucleophilic attack [61]. The energy gap between the analogous HOMO and LUMO orbitals Is called the HOMO-LUMO gap (ΔE), which could modulate the chemical reactivity, kinetic stability, and optical polarizability [62]. Thus, to examine the chemical reactivity and structural features of **2b** and **2e** compounds, DFT analysis was applied. Their HOMO and LUMO surface maps, energies observed for their HOMO (EHOMO) and LUMO (ELUMO) orbitals, and HOMO-LUMO energy gaps (ΔE), are displayed in Figure 5. 

Visualizing the HOMO and LUMO maps of **2b** and **2e** structures reveals that the regions of highest electron density (HOMO orbitals) almost exist over the tri-methoxy and di-methoxy phenyl fragments; the amide bonds also show a weak HOMO orbital association. These surfaces involving HOMO orbitals are highly favorable to associate with many valuable interactions within the ligand–protein interfaces such as hydrophobic interactions, π---σ and π---π stacking, and charge transfer interactions. On the other hand, mapping the LUMO orbitals reveals that the surface over the 4-F-phenyl-thiophene fragment is an electron-deficient surface as a result of the adjacent carbonyl-amide resonance and the high electronegative flour substituent. These both work as strong electron-withdrawing groups and led to minimizing the electron density within the thiophene ring. Despite this observed low electron density, the thiophene ring participated in a worthy π-cationic interaction with K-350 in both **2b** and **2e** compounds, based on its strong aromaticity. Due to these results, the finding of new thiophene carboxamide derivatives with higher thiophene electron density is expected to enhance the anti-cancer activity by boosting its interaction profile within the binding site. 

As summarized in Figure 5, the HOMO energy values of **2b** and **2e** candidates equaled −0.2013 and −0.2091 eV, whilst the LUMO energy values equaled −0.0681 and −0.0554 eV, respectively. As observed, the **2b** and **2e** molecules showed a very low HOMO-LUMO gap (ΔE) that equaled −0.13 and −0.15 eV, respectively. The higher energy gap of **2e** comp. indicates it has lower polarizability and a higher chemical hardness (needs more energy for excitation from HOMO to LUMO) compared to the **2b** compound.

**Figure 6:**
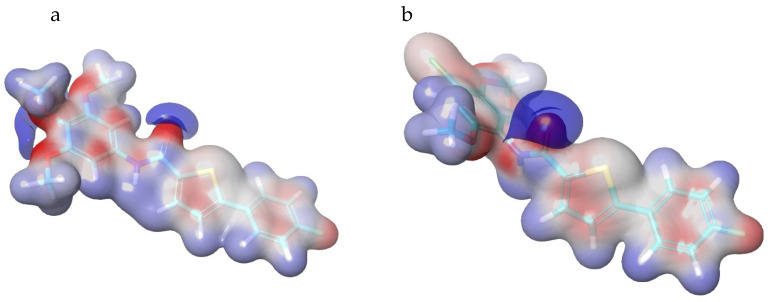
Electrostatic potential profiles of **2b** (**a**) and **2e** (**b**) compounds. The red color indicates high electro-negativity.

These observed features were supported by visualizing the electrostatic potential map (ESP) of both Compounds **2b** and **2e**, as shown in Figure 6. Within the ESP surface, the oxygen atoms of methoxy and carbonyl and the aromatic core are the regions of negative potential (red color), while the positive potentials (blue color) are present at the aromatic ring sides. The ESP gives a simple view of the interaction profile within the ligand–protein interface, especially at the primary stage, and is considered a valuable tool to predict the critical binding functional groups [63].

#### 3.4.3. Molecular Dynamics Simulations 

**Figure 7:**
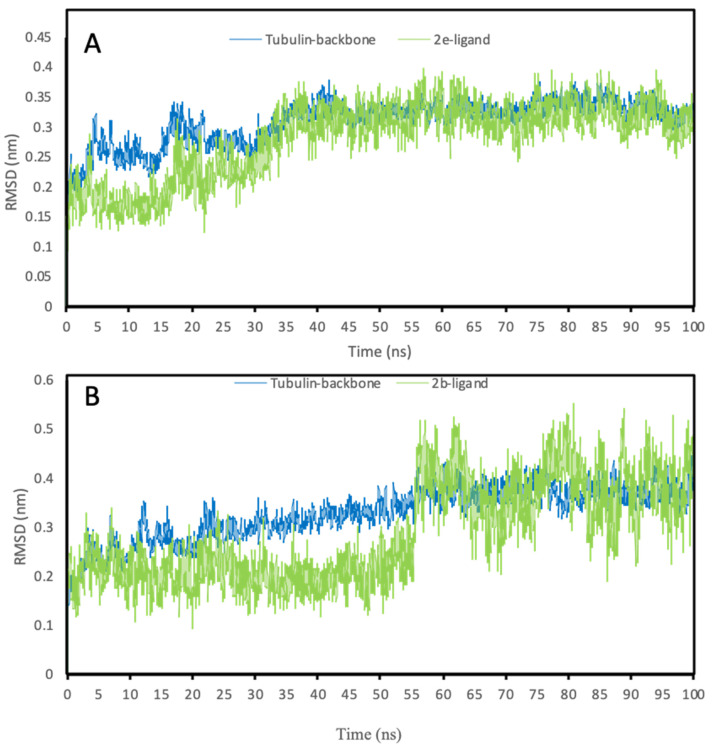
The time series related to RMSD values within the MD simulation trajectory. The RMSD plots for (**A**) 2b-tubulin complex and (**B**) 2e-tubulin complex at 300 K temperature for 100 ns of simulation trajectory. The figures represent the RMSD plots of proteins’ backbones (blue) and ligands (green).

The molecular dynamic (MD) simulation study aimed to realize the stability of ligand–protein complexes within the course of a certain time. The process of ligand fitting into the protein-binding pocket results in a conformational change in both ligand and protein backbones. These structural dynamics were simulated and various trajectories were generated and analyzed to get certain parameters such as the root mean square deviation (RMSD) plot, which is used to assess the compactness and stability of the whole ligand-target enzyme complexes [50]. The less RMSD values indicate less structural fluctuations, and observing an RMSD value less than 3 nm means that the ligand is optimally fitted and docked within the binding pocket and hence, is a ligand–protein complex of great stability. The best-docked poses of both **2b**- and **2e**-structures inside the colchicine-tubulin (PDB ID: 6XER) binding pocket were subjected to the MD simulations studies and thus, the molecular dynamics trajectories and conformational stability of these ligand–protein complexes were assessed using their obtained RMSD plots within a time scale of 100 ns. As presented in Figure 7, the protein (tubulin) backbone upon **2b** and **2e** ligands fitting shows trivial fluctuations (within 0.5 nm), and the simulation trajectories reached the plateau of equilibrium at approximately 50 ns in both complexes and then retained their stable state. Concerning ligand trajectories, the presented ligand-RMSD plots indicate that the **2b** candidate has a higher structural dynamicity than the **2e** structure within the binding pocket, especially after 50 ns. The **2b**-structure shows lower structural fluctuations and its plateau of equilibrium started at approximately 30 nm and retained its stable state. Both candidates show ideal simulation trajectories and minimal structural fluctuations (do not exceed 0.6 nm), which emphasize their high stability within the binding site.

**Figure 8:**
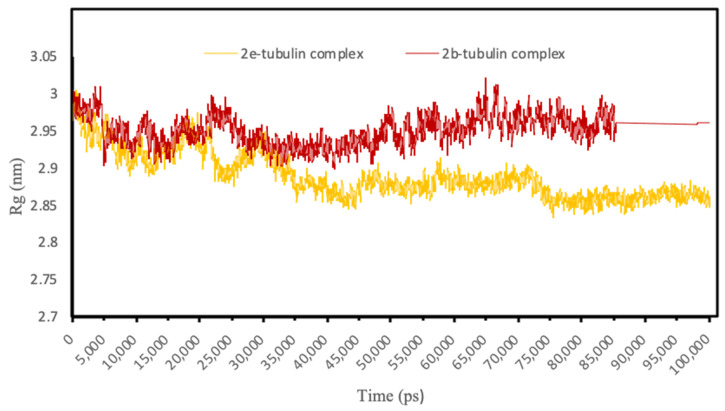
The radius of gyration plots for 2b-tubulin complex (red) and 2e-tubulin complex (yellow) for 100 ns of simulation trajectory at 300K temperature.

The radius of gyration (Rg) is another descriptor parameter applied to estimate the strength of macromolecular systems and ligand–protein complex structures. Based on the trajectories points, the Rg calculates the average distance between the center of mass and the axis of rotation. In terms of Rg, observing fewer fluctuations in the Rg values indicates better conformational stability of the ligand–protein complex, while getting a small Rg average value means more compactness. The Rg plots of **2b-** and **2e**-tubulin complexes are visualized in Figure 8. The Rg values within both complexes show minimal fluctuation ranges (within 0.2 nm) and low Rg average value (less than 3 nm) over a simulation trajectory of 100 ns and 300 K temperature, which is a good reference to their great stability and system compactness.

#### 3.4.4. ADME-T Analysis

Examining the thiophene carboxamide derivatives in terms of their predicted ADME-T properties showed the high druggability profiles of all the tested candidates (Table 3). This valuable tool comprised of testing a wide range and variety of physicochemical and pharmacokinetics parameters such as molecular weight (Mol._Mw); polar surface area (PSA), which is the van der waals surface area of oxygen, nitrogen, and carbonyl carbon atoms; computed the dipole moment of the molecule (Dipole), octanol/water partition coefficient (QPlogPo/w), octanol/gas partition coefficient (QplogPoct++), brain/blood partition coefficient, and their ability to cross the blood–brain barrier (QPlogBB); aqueous solubility (QPlogS); binding affinity to human serum albumin (QPlogKhsa); total solvent accessible surface area (SASA) in square angstroms using a probe with a 1.4 Å radius (SASA); Caco-2 cell permeability in nm/sec, which is a model for the gut–blood barrier (QPPCaco); number of likely metabolic reactions (#metab⁑) that indicate the susceptibility of the ligand for metabolic reactions; and finally to estimate the toxicity of the ligand via predicting the IC_50_ value for the blockage of HERG K+ channels. Blocking of the HERG K+ channel causes long QT syndrome, which is a life-threatening arrhythmia state. All the examined parameters were observed within the ideal ranges and did not exceed the recommended ranges. This study confirms the agreement of all evaluated candidates with the FDA-approved drugs that have potential drug-like properties with low toxicity.

## 4. Conclusions

To sum up, aiming to mimic the biological function and structural characteristics of the anti-cancer agent CA-4 compound, a group of novel thiophene carboxamide derivatives (**2a**–**2e**) was synthesized and tested against various cancer and normal cell lines to investigate their anti-cancer activity and safety profiles, respectively. As observed, all the synthesized compounds revealed considerable anticancer activity against the Hep3B cancer cell line, significantly **2b**, **2d**, and **2e** candidates. Therefore, the synthesized compounds represent newly successful CA-4 biomimetics, especially the substituted phenyl carboxamide part, which optimally simulated the structural geometry and polar surface area of CA-4 and hence the biological efficacy. This significant anti-cancer activity of thiophene carboxamide derivatives was accompanied by negligible toxicity profiles when tested against the two normal cell lines, besides demonstrating better cytotoxicity profiles than doxorubicin by at least 100-fold. On the other hand, investigating the ability of compound **2b** and **2e** compounds, as the most potent tested structures, to disturb the spheroids formation process within Heb3B cells, revealed that subjecting of these structures led to perturbing spheroid formation. This obtained disturbance in the spheroid formation, besides minimizing the size of aggregates formed, is supposed to play a key role in reducing cancer stemness, tumorigenicity, and cancer progression. Bioinformatics analysis explained the recorded biological potency of both **2b** and **2e** compounds and confirmed the existence of stable ligand–protein complexes. These examined complexes involved the formation of many favorable and unique interactions within the colchicine-tubulin-binding pocket. Also, these studies displayed that integrating the thiophene ring with a high aromaticity property resulted in improving the binding profiles via the formation of a new π-cationic interaction between the electron-rich thiophene ring and the cationic residue (K-350). Also, studying the molecular dynamics trajectory of **2b** and **2e** compounds within a time scale of 100ns at 300 K showed the formation of high stable and compacted ligand–protein complexes. All synthesized derivatives **2a**–**2e** obeyed Lipinski’s rule beside a wide variety of ADME-T parameters, which indicates that these structures agree with the FDA-approved drugs and are suitable to be utilized orally. In future investigations, we would like to follow all the recommendations mentioned here in our study and derive more novel thiophene-carboxamide analogs as promising anticancer agents. More studies in living organisms are needed to confirm these effects and figure out how to use these compounds to improve medicines.

## Figures and Tables

**Table 1:** IC_50_ (µM) of phenyl-thiophene-carboxamide compounds (**2a**–**2e**) on several cell lines.

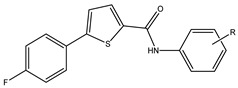						
IC_50_ (µM)						
Cell line	2a	2b	2c	2d	2e	DOX
R	H	3,4,5-tri-MeO	4-*t*-butyl	3,5-di-MeO	2,5-diMeO-4-CL	-
Hep3B	28.02 ± 2.05	5.46 ± 1.47	189.43 ± 2.78	8.85 ± 1.45	12.58 ± 2.57	1.21 ± 1.0
B16F1	Ni	Ni	Ni	Ni	112.11 ± 1.99	<1
Colo205	Ni	35.08 ± 2.05	Ni	Ni	74.82 ± 1.45	<1
HepG2	283.67 ± 1.79	>300	>300	>300	18.19 ± 0.78	1.598 ± 0.67
CaCo-2	Ni	Ni	Ni	Ni	82.89 ± 2.45	<1
HeLa	>300	205.33	>300	>300	39.58 ± 1.88	1.55 ± 1.35
MCF-7	>300	29.74 ± 2.74	Ni	>300	42.50 ± 2.59	<1
LX-2	Ni	Ni	Ni	Ni	Ni	<1
Hek293t	Ni	278.13 ± 2.75	Ni	Ni	105.23 ± 1.08	1.24 ± 0.87

Note: *p* value ≤ 0.05. Ni: no inhibition.

**Table 2:** The interaction pattern, docking scores, and MM-GBSA values of colchicine, CA-4, **2b**, and **2e** compounds within tubulin-binding pocket (6XER).

Name	H. Bs	π-Cation	HPHO	Docking Score	MM-GBSA (ΔG)
**2b**	C:ASN 101	D:LYS 350	D:LEU 248, D: LEU 255, D: ALA 316	−6.13	−36.02
**2e**	D:GLN 245, C:SER 178	D:LYS 350	D:LEU 246, D: ALA 148, D:LYS 252, D:LYS 350	−6.46	−41.47
**Colchicine**	C:VAL 181, C:ASN 101, D:LYS 350		D:ALA 248, D:LYS 252, D:LEU 253, D:ASN 256, D:LYS 350,	−9.23	−42.75
**CA-4**	D:CYS 239, C:VAL 181		D:LEU 246, D:LEU 253, D:ASN 256	−8.29	−46.72

H. Bs: hydrogen bond interactions. π-Cation: pi-cationic interactions. HPHO: hydrophobic interactions.

**Table 3:** The ADME-T analysis.

	Mol._Mw	PSA	Percent Human Oral Absorption	Dipole	QPlogPo/w	QPlogPoct++	QPlogBB	QPlogS	QPlogKhsa	SASA	FOSA	FISA	PISA	WPSA	Volume	QPpolrz	QPPCaco	#metab⁑	QPlogHERG	Rule of three	Rule of five
2a	297.3	35.6	100	5.66	4.5	14.7	0.17	−5.2	0.60	558.7	0.0	47.5	433.5	77.6	939.0	34.28	3510	2	−6.3	0	0
2e	391.8	50.02	100	6.16	5.3	17.4	0.20	−6.6	0.78	666.0	183.0	45.6	288.3	149.0	1146.6	39.9	3659	3	−6.2	1	1
2d	357.3	52.10	100	6.01	4.8	16.5	0.04	−5.8	0.63	632.7	183.5	45.8	325.5	77.8	1090.5	38.0	3641	4	−6.1	1	0
2c	353.4	35.7	100	5.70	5.8	16.9	0.08	−6.4	1.14	660.2	192.8	47.5	342.0	77.8	1157.7	41.3	3508	1	−6.3	1	1
2b	387.4	58.9	100	6.00	4.9	17.7	−0.02	−6.2	0.67	674.2	266.2	45.4	284.5	77.9	1177.9	40.4	3669	4	−6.1	1	0
Rec. Values	130–725	7–200	>80% high <25% low	1.0–12.5	-2.0–6.5	8.0–35	-3–1.2	-6.0–0.5	-1.5–1.5	300–1000	0.0–750.0	7.0–330.0	0.0–450.0	0.0–175.0	500–2000	13.0–70.0	<25 poor >500 great	1–8	Below −5	<3	<4

The ADME-T properties of the thiophene carboxamide derivatives using QiKProp module (schrödinger 12.1, LLC., New York, NY, USA) running in normal mode. Mol Mw: molecular weight of the molecule. PSA: van der Waals surface area of polar nitrogen, oxygen atoms, and carbonyl carbon atoms. SASA: total solvent accessible surface area (SASA) in square angstroms using a probe with a 1.4 Å radius. FOSA: Hydrophobic component of the SASA (saturated carbon and attached hydrogen). FISA: Hydrophilic component of the SASA (SASA on N, O, H on heteroatoms, carbonyl C). PISA: π (carbon and attached hydrogen) component of the SASA. WPSA: Weakly polar component of the SASA (halogens, P, and S). Volume: Total solvent-accessible volume in cubic angstroms using a probe with a 1.4 Å radius. QPpolrz: Predicted polarizability in cubic angstroms. QPPCaco: predicted apparent Caco-2 cell permeability in nm/sec. Percent Human Oral Absorption: predicted human oral absorption on a 0–100% scale. #metab⁑: number of likely metabolic reactions. Dipole: computed dipole moment of the molecule. QPlogPo/w: predicted octanol/water partition coefficient. QPlogPoct++: predicted octanol/gas partition coefficient. QPlogBB: predicted brain/blood partition coefficient. QPlogHERG: Predicted IC_50_ value for blockage of HERG K+ channels. QPlogS: predicted aqueous solubility, S in mol dm−3. QPlogKhsa: prediction of binding to human serum albumin. Rule of three: number of violations of Jorgensen’s rule of 3. Rule of five: number of violations of Lipinski rule of 5.

## Data Availability

Not applicable.
